# The association between region of birth and sexually transmitted infections among people of black Caribbean ethnicity attending sexual health services in England, 2015

**DOI:** 10.1371/journal.pone.0228654

**Published:** 2020-02-21

**Authors:** Ana K. Harb, Hamish Mohammed, Martina Furegato, Sonali Wayal, Catherine H. Mercer, Gwenda Hughes

**Affiliations:** 1 National Infection Service, Public Health England, London, United Kingdom; 2 Centre for Population Research in Sexual Health and HIV, University College London, London, United Kingdom; 3 The Applied Diagnostic Research and Evaluation Unit (ADREU), St George’s, University, City, London, United Kingdom; 4 National Institute for Health Research Health Protection Research Unit in Blood Borne and Sexually Transmitted Infections, University College London, London, United Kingdom; University of Pretoria, SOUTH AFRICA

## Abstract

**Background/Introduction:**

In England, people of Black Caribbean (BC) ethnicity are disproportionately affected by sexually transmitted infections (STIs), but it is unclear whether this varies by their region of birth.

**Aim(s)/Objectives:**

To examine differences in STI diagnoses among UK- and Caribbean-born BC people.

**Methods:**

Data on STI diagnoses in BC people attending specialist sexual health services (SHSs) during 2015 and living in England were obtained from the GUMCAD STI surveillance system, the national surveillance system for STIs in England. Associations between being UK- or Caribbean-born and each of several STI diagnoses were examined, using univariate and multivariable generalised estimated equations logistic regression models adjusted for sexual orientation, place of residence (London vs. non-London), HIV status, area-level deprivation, and STI diagnosis in the last year. All analyses were stratified by age (<25 vs. ≥25 years).

**Results:**

In 2015, 63,568 BC people made 108,881 attendances at specialist SHSs; 81.9% of these attendances were made by UK-born BCs. The median age (years) was 26 for UK-born and 35 for Caribbean-born people (p≤0.001). Chlamydia, gonorrhoea and non-specific genital infection (NSGI) were the most commonly diagnosed STIs among UK- (5.8%, 2.1% and 2.8%) and Caribbean-born people (4.5%, 1.7% and 3.5%) respectively. Among BCs aged under 25, no significant differences in STIs were found between UK- and Caribbean-born people. Among BCs aged ≥25, compared to Caribbean-born people, those who were UK-born were more likely to be diagnosed with chlamydia (AOR 1.15 [95%C.I. 1.04–1.27]); gonorrhoea (AOR 1.23 [95%C.I. 1.06–1.45]) and genital herpes (AOR 1.23 [95% C.I. 1.10–1.56]) and less likely to be diagnosed with NSGI (AOR 0.89 [95% C.I. 0.80–0.99]) and Trichomoniasis (AOR 0.84 [95% C.I. 0.71–0.99]).

**Discussion/Conclusion:**

STI diagnoses in BC people aged ≥25 attending specialist SHSs vary by region of birth. Country of birth may have an influence on social and sexual networks and therefore transmission of STIs.

## Introduction

Black ethnic minorities bear a disproportionate burden of sexually transmitted infections (STIs) in many high-income countries[1]. In the UK, the highest diagnosis rates of STIs are among people of black Caribbean (BC) ethnicity[2]. These high rates are a consequence of the interaction of cultural, socioeconomic and sexual behavioural characteristics[3–5], and these are likely to vary depending on an individual’s region of birth.

The BC (i.e. Afro-Caribbean or African Caribbean) ethnic group includes people of African ancestral origins whose family settled in the Caribbean before emigrating to the UK[6]. BC people are a diverse population from at least two perspectives: first, they are from a number of different countries in the Caribbean, each with their own sociocultural influences[7] and, second, they comprise people born in the Caribbean who migrated some decades ago through to sixth-generation people who identify as BC, but who were born in the UK[8,9]. Prevalence of some STIs by region of birth have been described higher in the Americas than in Western Europe[10]. The extent to which region of birth is associated with the risk of STIs is unclear. Thus, we examined differences in the likelihood of being diagnosed with STIs between UK- and Caribbean-born BC people attending specialist sexual health services (SHSs) in England.

## Methods

Data on STI diagnoses in BC people living in England were obtained for 1 January– 31 December 2015 from the GUMCAD STI surveillance system, the mandatory national surveillance system for STIs in England (formerly known as Genitourinary Medicine Clinic Activity Dataset). GUMCAD is a patient-level dataset containing information on STI diagnoses and services provided by all specialist SHSs in England, as well as key sociodemographic data, including country of birth[11]. Specialist SHSs refers to genitourinary medicine (GUM) and integrated GUM/sexual and reproductive health (SRH) services. Attendances made by people who self-identified as BC but had unknown region of birth (14.2%) or were born outside of the Caribbean or the UK (4.3%) were excluded from the analysis, as were those of unknown gender (0.01%), unknown sexual orientation (2.0%) or living outside England (2.0%).

For this analysis, the following countries and territories were defined as being in the Caribbean: Antigua and Barbuda, Aruba, the Bahamas, Barbados, Cuba, Dominica, Dominican Republic, Grenada, Haiti, Jamaica, Netherland Antilles, Saint Kitts and Nevis, Saint Lucia, Saint Vincent and the Grenadines, and Trinidad and Tobago.

### Clinical definitions

All STIs reported in GUMCAD were confirmed diagnoses, defined using national STI management guidelines[12], that were coded and reported as such.

The list of STIs reported in history of being diagnosed in the last year with any STI includes *Chlamydia trachomatis*, *Haemophilus ducreyi* (Chancroid), *Klebsiella granulomatis* (Donovanosis), herpes simplex virus (Genital herpes: First episode), human papillomavirus (Genital warts: First episode), *Neisseria gonorrhoeae*, serovars L1, L2, L2a, L2b or L3 of *Chlamydia trachomatis* that cause Lymphogranuloma venereum (LGV), *Mycoplasma genitalium*, Molluscum contagiosum, Non-specific genital infection (NSGI), *Pthirus pubis* (Pediculosis pubis), Pelvis Inflammatory Disease (PID) & epididymitis: Unspecified aetiology, *Sarcoptes scabiei* (Scabies), *Treponema pallidum* (Syphilis: Primary Syphilis: Secondary Syphilis: Early latent) and *Trichomonas vaginalis* (Trichomoniasis). The diagnosis code used to report NSGI should include complicated and uncomplicated cases. For males, cases of NSGI are recorded in the presence of polymorphonuclear leucocytes (at >5 per high power field) and in the absence of laboratory confirmed C*hlamydia trachomatis* and *Neisseria gonorrhoeae*. Females being treated for non-specific mucopurulent cervicitis are also reported as NSGI.

### Data analysis

Based on the Kolmogorov-Smirnov test[13], the age distribution of BC attendees varied by region of birth, therefore all models were stratified by age, using 25 years as a cut-off due to the higher STI diagnosis rates within people under 25 year olds[2].

Separate models were run for <25 and ≥25 year old UK-born compared with Caribbean-born BC attendees to calculate the unadjusted odds ratio (OR) and adjusted odds ratios (AOR) of being diagnosed with each STI. In GUMCAD, a patient is permitted only one record relating to a particular STI in a six week period; repeat codes for the same STI within this period are considered as relating to the same episode of care and so are removed to prevent over-counting of diagnoses. The following STIs were included in the analysis: Chlamydia; gonorrhoea; NSGI; genital herpes (1st episode); HIV; genital warts (1st episode) and trichomoniasis. The following were considered as confounders: gender and male sexual orientation[14] (coded heterosexual male, men who have sex with men, or women), place of residence (London vs. non-London) [15], HIV status[16] (coded negative or unknown vs known positive), a history of STI diagnosis in the last year[17] (coded yes or no), and area-level socioeconomic deprivation defined using quintiles of the Index of Multiple Deprivation (IMD)[18] for each lower super output area (LSOA) of residence.

As this was an attendance-level analysis and many attendees had multiple attendances, all univariate and multivariable models were built using generalised estimated equations logistic regression models to account for these clustered observations.

## Results

In 2015, among those with a known region of birth, 63,568 BC people made 108,881 attendances at specialist SHSs in England. These attendances were 3.9% of the total attendances that year (2,780,434). Of these 108,881 attendances by BC people, the majority (81.9%) were by UK-born BC people.

The median age of UK- and Caribbean-born BC people was 26 (min:15—max: 83) and 35 (min:15—max: 91; (p<0.001)) years, respectively. Fewer UK-born (52.0%) vs. Caribbean-born (64.3%; p<0.001) BC people resided in London. For both UK- and Caribbean-born people, STIs were most likely to be diagnosed in those living in the most socioeconomically deprived areas of England.

Chlamydia, gonorrhoea and NSGI were the most commonly reported STIs among UK-born (5.8%, 2.1% and 2.8%) and Caribbean-born BC people (4.5%, 1.7% and 3.5%) respectively, while the fourth most common infection was genital warts (1.5%) for UK-born BC people and trichomoniasis for Caribbean-born BC people (1.3%). HIV was less commonly diagnosed at attendances by UK-born (0.1%) vs. Caribbean-born BC people (0.2%).

For those <25 years old (Table 1), and diagnosed with chlamydia, gonorrhoea, NSGI, genital herpes, or genital warts, a higher proportion of Caribbean-born people were London residents compared to UK-born BCs. Also, a higher proportion of Caribbean-born BC people with a history of STI diagnosis in the last year were diagnosed with chlamydia compared to UK-born BC people.

**Table 1:** Percentage of all attendances by black Caribbean people at specialist sexual health services resulting in STI diagnoses and differences in socio-demographic profile by region of birth among those aged <25 years^a^, 2015.

	Chlamydia				Gonorrhoea				NSGI				Trichomoniasis				Genital Herpes				HIV—newly diagnosed				Genital warts			
	UK- born		Caribbean-born		UK- born		Caribbean-born		UK- born		Caribbean-born		UK- born		Caribbean-born		UK- born		Caribbean-born		UK- born		Caribbean-born		UK- born		Caribbean-born	
	n	%	n	%	n	%	n	%	n	%	n	%	n	%	n	%	n	%	n	%	n	%	n	%	n	%	n	%
**Diagnoses**	**3,116**	**8.0%**	**334**	**8.8%**	**941**	**2.4%**	**107**	**2.8%**	**770**	**2.0%**	**93**	**2.4%**	**392**	**1.0%**	**46**	**1.2%**	**509**	**1.9%**	**33**	**0.9%**	**15**	**0.0%**	**3**	**0.1%**	**807**	**2.1%**	**69**	**1.8%**
**Age median (min—max)**	20 (15–24)		21 (15–24)		20 (15–24)		21 (15–24)		22 (15–24)		22 (16–24)		20 (15–24)		20 (16–24)		21 (15–24)		22 (16–24)		21 (17–24)		22 (22–23)		20 (15–24)		20 (16–24)	
**Gender and male sexual orientation**																												
Heterosexual men^b^	1,351	43.3%	157	47.0%	355	37.7%	53	49.5%	680	88.3%	85	91.4%	18	4.6%	3	6.5%	177	34.8%	8	24.2%	3	20.0%	0	0.0%	373	46.2%	31	44.9%
Men who have sex with men	86	2.8%	15	4.5%	177	18.8%	19	17.8%	30	3.9%	5	5.4%	0	0.0%	0	0.0%	9	1.8%	1	3.0%	8	53.3%	3	100.0%	25	3.1%	3	4.3%
Women	1,679	53.9%	162	48.5%	409	43.5%	35	32.7%	60	7.8%	3	3.2%	374	95.4%	43	93.5%	323	63.4%	24	72.8%	4	26.7%	0	0.0%	409	50.7%	35	50.8%
**Resident in London**																												
No^b^	**1,659**	**53.2%**	**135**	**40.4%**	**449**	**47.7%**	**28**	**26.2%**	**337**	**43.7%**	**31**	**33.3%**	180	46.0%	16	34.8%	**295**	**58.0%**	**11**	**33.3%**	7	46.7%	1	33.3%	**476**	**59.0%**	**29**	**42.0%**
Yes	**1,457**	**46.8%**	**199**	**59.6%**	**492**	**52.3%**	**79**	**73.8%**	**433**	**56.3%**	**62**	**66.7%**	212	54.0%	30	65.2%	**214**	**42.0%**	**22**	**66.7%**	8	53.3%	2	66.7%	**331**	**41.0%**	**40**	**58.0%**
**History of STI**^c^ **in the last year**																												
No^b^	**2,598**	**83.4%**	**263**	**78.7%**	**77**	**72.0%**	**763**	**83.1%**	561	72.9%	65	69.9%	292	74.5%	34	73.9%	442	86.8%	27	81.8%	14	93.3%	2	66.7%	715	88.6%	56	81.2%
Yes	**518**	**16.6%**	**71**	**21.3%**	**30**	**28.0%**	**178**	**18.9%**	209	27.1%	28	30.1%	100	25.5%	12	26.1%	67	13.2%	6	18.2%	1	6.7%	1	33.3%	92	11.4%	13	18.8%
**HIV status**																												
Negative or unknown^b^	3,112	99.9%	334	100.0%	922	98.0%	107	100.0%	768	99.7%	93	100.0%	389	99.2%	46	100.0%	508	99.8%	33	100.0%	-	-	-	-	807	100.0%	68	98.5%
Known positive	4	0.1%	0	0.0%	19	2.0%	0	0.0%	2	0.3%	0	0.0%	3	0.8%	0	0.0%	1	0.2%	0	0.0%	-	-	-	-	0	0.0%	1	1.5%
**Residential area-level deprivation 2015**^d^																												
1 (most deprived)^b^	1,284	43.7	142	45.1%	434	48.2%	48	46.1%	288	39.1%	42	47.2%	191	50.4%	25	56.8%	185	38.1%	16	50.0%	8	53.4%	2	66.7%	298	39.1%	30	45.5%
2	861	29.3	104	33.0%	252	28.0%	32	30.8%	229	31.1%	31	34.8%	109	28.7%	10	22.7%	150	30.9%	9	28.2%	5	33.3%	1	33.3%	216	28.3%	21	31.8%
3	453	15.4	41	13.0%	122	13.5%	17	16.3%	142	19.3%	11	12.4%	55	14.5%	7	15.9%	77	15.9%	3	9.4%	0	0.0%	0	0.0%	130	17.1%	9	13.7%
4	204	6.9	13	4.1%	63	7.0%	4	3.9%	55	7.4%	4	4.5%	20	5.3%	0	0.0%	45	9.3%	2	6.2%	0	0.0%	0	0.0%	68	8.9%	3	4.5%
5 (least deprived)	139	4.7	15	4.8%	30	3.3%	3	2.9%	23	3.1%	1	1.1%	4	1.1%	2	4.6%	28	5.8%	2	6.2%	2	13.3%	0	0.0%	50	6.6%	3	4.5%

^a^The list of countries and territories included in the Caribbean region were Antigua and Barbuda, Aruba, The Bahamas, Barbados, Cuba, Dominica, Dominican Republic, Grenada, Haiti, Jamaica, Netherland Antilles, Saint Kitts and Nevis, Saint Lucia, Saint Vincent and the Grenadines, and Trinidad and Tobago.

^b^First line is the baseline of the analysis.

^c^History of being diagnosed in the last year with any STI (Chlamydia, Chancroid, Donovanosis, Genital herpes: First episode, Genital warts: First episode, Gonorrhoea, LGV, Mycoplasma genitalium, Molluscum contagiosum, Non-pecific genital infection, Pediculosis pubis, PID & epididymitis: Unspecified, Scabies, Syphilis: Primary Syphilis: Secondary Syphilis: Early latent and Trichomoniasis).

^d^Residential area-level deprivation is defined using the Index of Multiple Deprivation (IMD) for each lower super output area (LSOA) of residence. LSOAs are small areas designed to be of a similar population size, with an average of approximately 1,500 residents or 650 households.

https://www.gov.uk/government/statistics/english-indices-of-deprivation-2015.

IMD was not available for those diagnoses reported with LSOAs older than the 2011 Census Super Outputs Areas.

In bold, associations statistically significant (p<0.05).

For those ≥ 25 years old (Table 2), and diagnosed with gonorrhoea or trichomoniasis, a higher proportion of those Caribbean-born were London residents compared to UK-born BC people.

**Table 2:** Percentage of all attendances by black Caribbean people at specialist sexual health services resulting in STI diagnoses and differences in socio-demographic profile by region of birth among those aged ≥25 years^a^, 2015.

	Chlamydia				Gonorrhoea				NSGI				Trichomoniasis				Genital Herpes				HIV—newly diagnosed				Genital warts				
	UK- born		Caribbean-born		UK- born		Caribbean-born		UK- born		Caribbean-born		UK- born		Caribbean-born		UK- born		Caribbean-born		UK- born		Caribbean-born		UK- born		Caribbean-born	
	n	%	n	%	n	%	n	%	n	%	n	%	n	%	n	%	n	%	n	%	n	%	n	%	n	%	n	%
**Diagnoses**	**2,096**	**4.2%**	**556**	**3.5%**	**932**	**1.9%**	**235**	**1.5%**	**1,698**	**3.4%**	**590**	**3.7%**	**640**	**1.3%**	**221**	**1.4%**	**646**	**1.3%**	**157**	**1.0%**	**51**	**0.1%**	**27**	**0.2%**	**516**	**1.0%**	**113**	**0.7%**
**Age median (min—max)**	29 (25–59)		34 (25–79)		30 (25–58)		36 (25–76)		32 (25–78)		40 (25–83)		34 (25–65)		40 (25–82)		30 (25–67)		38 (25–74)		35 (25–58)		11 (25–73)		29 (25–59)		36 (25–74)	
**Gender and male sexual orientation**																												
Heterosexual men^b^	354	63.7%	1,223	58.4%	**381**	**40.9%**	**111**	**47.2%**	1,546	91.1%	541	91.7%	**82**	**12.8%**	**46**	**20.8%**	271	41.9%	72	45.9%	11	21.6%	13	48.1%	301	58.4%	71	62.8%
Men who have sex with men	59	10.6%	208	9.9%	**361**	**38.7%**	**92**	**39.1%**	89	5.2%	29	4.9%	**1**	**0.2%**	**0**	**0.0%**	14	2.2%	8	5.1%	29	56.8%	10	37.0%	25	4.8%	4	3.6%
Women	143	25.7%	665	31.7%	**190**	**20.4%**	**32**	**13.6%**	63	3.7%	20	3.4%	**557**	**87.0%**	**175**	**7.2%**	361	55.9%	77	49.0%	11	21.6%	4	14.9%	190	36.0%	38	33.6%
**Resident in London**																												
No^b^	1,057	50.4%	205	36.9%	**389**	**41.7%**	**78**	**33.2%**	604	35.6%	170	28.8%	**310**	**48.4%**	**85**	**38.5%**	273	42.3%	58	36.9%	17	33.3%	12	44.4%	239	46.3%	41	36.3%
Yes	1,039	49.6%	351	63.1%	**543**	**58.3%**	**157**	**66.8%**	1,094	64.4%	420	71.2%	**330**	**51.6%**	**136**	**61.5%**	373	57.7%	99	63.1%	34	66.7%	15	55.6%	277	53.7%	72	63.7%
**History of STI**^**c**^ **in the last year**																												
No^b^	1,747	83.3%	477	85.8%	704	75.5%	189	80.4%	1,216	71.6%	428	72.5%	547	85.5%	189	85.5%	578	89.5%	140	89.2%	42	82.3%	23	85.2%	476	92.2%	105	92.9%
Yes	349	16.7%	79	14.2%	228	24.5%	46	19.6%	482	28.4%	162	27.5%	93	15.5%	32	14.5%	68	10.5%	17	10.8%	9	17.7%	4	14.7%	40	7.8%	8	7.1%
**HIV status**																												
Negative or unknown^b^	2,010	95.9%	520	93.5%	798	85.6%	195	83.0%	1,678	98.8%	577	97.8%	638	99.7%	220	99.5%	**639**	**98.9%**	**151**	**96.2%**					509	98.6%	111	98.2%
Known positive	86	4.1%	36	6.5%	134	14.4%	40	17.0%	20	1.2%	13	2.2%	2	0.3%	1	0.5%	**7**	**1.1%**	**6**	**3.8%**					7	1.4%	2	1.8%
**Residential area-level deprivation 2015**^d^	921	46.2%	269	50.7%	88	38.8%	414	46.1%	663	40.7%	250	43.7%	325	53.6%	133	63.3%	262	42.4%	69	46.0%	19	38.8%	6	26.1%	191	39.1%	45	42.5%
1 (most deprived)^b^	588	29.5%	157	29.6%	72	31.7%	260	28.9%	559	34.3%	175	30.6%	183	30.2%	54	25.7%	174	28.2%	44	29.3%	17	34.7%	11	47.8%	151	30.9%	37	34.9%
2	295	14.7%	79	14.9%	49	21.6%	137	15.3%	237	14.6%	101	17.7%	69	11.4%	18	8.6%	107	17.3%	23	15.3%	8	16.3%	6	26.1%	78	15.9%	15	14.1%
3	144	7.2%	8	1.5%	12	5.3%	59	6.6%	116	7.1%	34	5.9%	21	3.5%	4	1.9%	49	7.9%	9	6.0%	4	8.2%	0	0.0%	50	10.2%	6	5.7%
4	48	2.4%	18	3.4%	6	2.6%	28	3.1%	53	3.3%	12	2.1%	8	1.3%	1	0.5%	26	4.2%	5	3.4%	1	2.0%	0	0.0%	19	3.9%	3	2.8%
5 (least deprived)																												

^a^The list of countries and territories included in the Caribbean region were Antigua and Barbuda, Aruba, The Bahamas, Barbados, Cuba, Dominica, Dominican Republic, Grenada, Haiti, Jamaica, Netherland Antilles, Saint Kitts and Nevis, Saint Lucia, Saint Vincent and the Grenadines, and Trinidad and Tobago.

^b^First line is the baseline of the analysis.

^c^History of being diagnosed in the last year with any STI (Chlamydia, Chancroid, Donovanosis, Genital herpes: First episode, Genital warts: First episode, Gonorrhoea, LGV, Mycoplasma genitalium, Molluscum contagiosum, Non-pecific genital infection, Pediculosis pubis, PID & epididymitis: Unspecified, Scabies, Syphilis: Primary Syphilis: Secondary Syphilis: Early latent and Trichomoniasis).

^d^Residential area-level deprivation is defined using the Index of Multiple Deprivation (IMD) for each lower super output area (LSOA) of residence. LSOAs are small areas designed to be of a similar population size, with an average of approximately 1,500 residents or 650 households.

https://www.gov.uk/government/statistics/english-indices-of-deprivation-2015.

IMD was not available for those diagnoses reported with LSOAs older than the 2011 Census Super Outputs Areas.

In bold, associations statistically significant (p<0.05).

**Fig 1:**
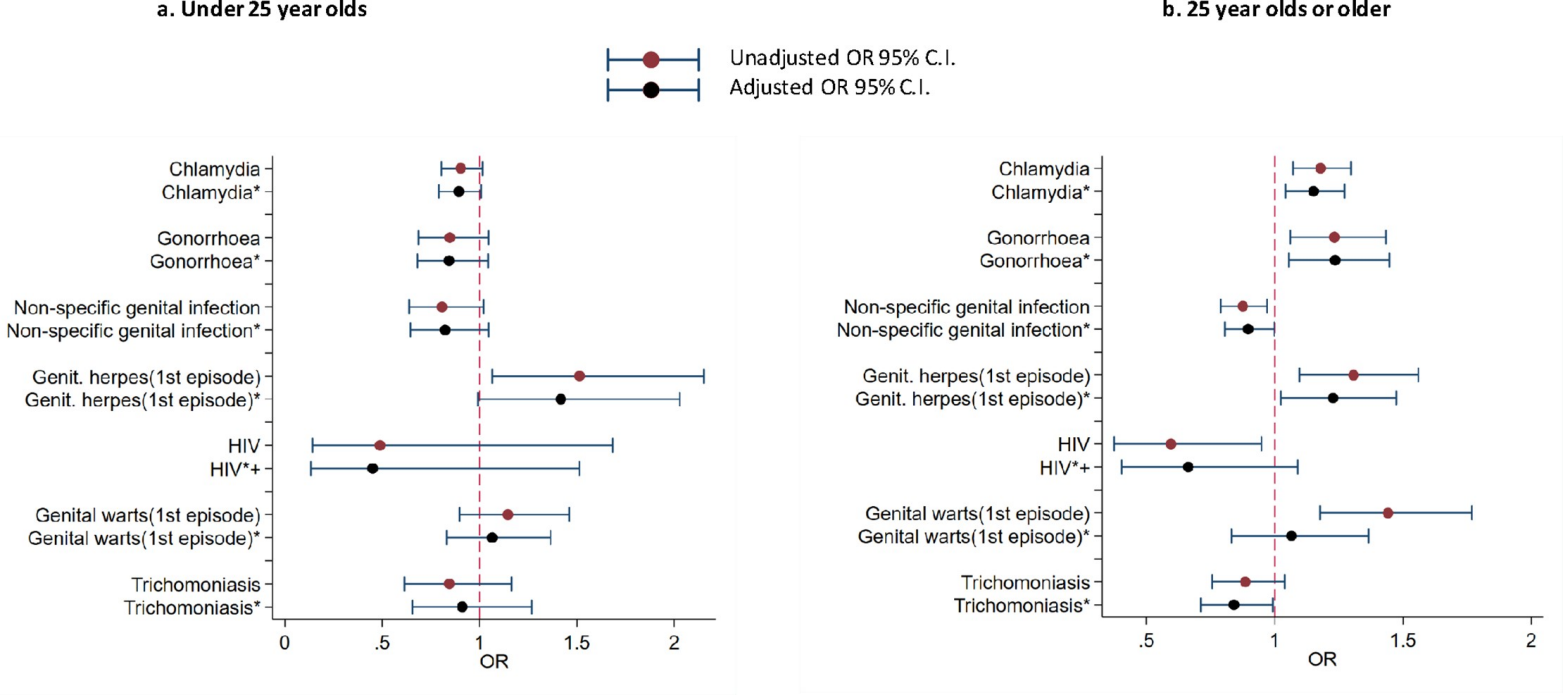
Unadjusted and adjusted associations with being diagnosed with selected STIs by region of birth and age-group, England, 2015. ^1^The reference group (1) is Caribbean-born. *Adjusted for gender/sexual-orientation, London residence, area-level deprivation, history of STI diagnosis in the last year and HIV status. ^+^Not adjusted for HIV status.

The unadjusted and factors adjusted associations between diagnosis with selected STIs and attendances by UK- vs. Caribbean-born BC people at specialist sexual health services, by age-group, England, 2015 are shown in Fig 1.

Among BC attendees <25 years, those who were UK-born were more likely to be diagnosed with genital herpes (OR 1.51 [95%C.I. 1.06–2.15]) than Caribbean-born people. However, after adjusting for confounders, this association did not remain significant.

According to the univariate models, among those aged ≥25 years, UK-born people were more likely to be diagnosed with chlamydia (1.17 [1.07–1.30]); gonorrhoea (1.23 [1.06–1.43]); genital herpes (1.30 [1.10–1.56]) and genital warts (1.44 [1.18–1.77]), and less likely to be diagnosed with NSGI (0.87 [0.79–0.97]) and HIV (0.59 [0.37–0.95]) compared to Caribbean-born people.

In the multivariable analysis, the association with region of birth remained statistically significant for all but two of these STIs (genital warts and HIV) with UK-born BC people being more likely to be diagnosed with chlamydia (1.15 [95%C.I. 1.04–1.27]); gonorrhoea (1.23 [1.06–1.45]) and genital herpes (1.23 [1.02–1.47]), and less likely to be diagnosed with NSGI (0.89 [0.80–0.99]) and trichomoniasis (0.84 [0.71–0.99]) compared to Caribbean-born people.

## Discussion

In this analysis, we examined the differences in the likelihood of STI diagnoses among BC people by region of birth. While BC people have previously been shown to have higher STI diagnosis rates[19], we found that the likelihood of being diagnosed with STI varies markedly by region of birth among those aged over 25 years old. UK-born BC people were more likely to be diagnosed with the most commonly diagnosed STIs in England, i.e. chlamydia, gonorrhoea, genital herpes, while their Caribbean-born counterparts were more likely to be diagnosed with trichomoniasis and NSGI. The reasons for this are unknown but may be associated with differences in patterns of health-seeking behaviours, and/ or differences in their sexual networks[20,21].

There is evidence of differences in sexual health outcomes of UK-born compared to migrant populations. For example, a large proportion of all HIV cases among heterosexuals in most countries of the European Union originate from outside of Europe[22]. In England, black ethnic minorities and especially black Caribbean populations are at much greater risk of STIs, especially for gonorrhoea and trichomoniasis[19]. However, there is limited published evidence specifically for STIs in BC people living in England, and this was not differentiated by region of birth[23].

In our analysis, adjusting for confounders had very little impact on the ORs apart from for genital warts in those aged ≥25. It is interesting to note that the direction of association between region of birth and chlamydia and gonorrhoea diagnoses differed by age-group. This might suggest that the sexual networks of UK-born vs. Caribbean-born BCs may be similar until the age of 25 and then changes occur in BCs aged ≥25 potentially due to change in partnership type and sexual mixing patterns, considered key determinants of STI transmission, with respect to demographic characteristics, general health, health behaviours and sexual history[24]. This also could be due to the length of time those born in the Caribbean had spent in the UK (i.e. those <25 years may have, on average, spent less time in the UK and might have had Relationship and Sexual Education in the UK with different sexual health outcomes compared to those who were older). Further investigation of behavioural factors may explain the differences in STI risk by region of birth. The potential role of partnership concurrency in maintaining high rates of bacterial STIs in BC populations has been documented[25–27]. It is possible that the differences in the risk of STIs by region of birth are related to different background prevalence of different STIs in different countries[10], and perhaps Caribbean-born who are residents in the UK travel more frequently to the Caribbean than the UK-born BC people, therefore more likely to be exposed there.

While GUMCAD is vast in scale and includes all attendances made at specialist SHSs in England, a limitation is that it does not yet collect detailed data on sexual behaviour (e.g. condom use, number of partners, or partnership concurrency), as well as broader health-related factors, such as alcohol and drug use, which may confound the observed associations. Furthermore, there is no information on factors that might play a role in the risk of STIs such as the length of time Caribbean-born people have lived in the UK and their frequency of travel to the Caribbean.

Historically, many Caribbean-born people were of working age when they migrated with their children to the UK after the British Nationality Act of 1948[28,29]. This pattern of migration may have changed over time[30], and differences between those migrating to the UK as a child or an adult might help to explain differences in STI risk factors. This information has been collected as part of the work of the National Institute for Health Research Health Protection Research Unit in Blood Borne and Sexually Transmitted Infections[31] and will be explored in future analyses. Another limitation of this study is that, despite the socio-cultural differences and varying levels of development that exist among Caribbean countries, all Caribbean-born people were included in a single category for analysis as there were insufficient observations for each of the 15 countries and territories considered in this group.

This study offers insights into disparities in STIs by region of birth among BC people living in England. These disparities are partially explained by differences in demographics between UK- and Caribbean-born people but also explained by region of birth. However, future research should examine behavioural differences, including the role of sexual networks, concurrency, partnership type and numbers, condom use, and sexual healthcare-seeking behaviour to better understand ethnic disparities in STI diagnoses.
